# Midazolam, hippocampal function, and transitive inference: Reply to Greene

**DOI:** 10.1186/1744-9081-4-5

**Published:** 2008-01-30

**Authors:** Michael J Frank, Randall C O'Reilly, Tim Curran

**Affiliations:** 1 Dept of Psychology and Program in Neuroscience, University of Arizona, Tucson, USA; 2 Dept of Psychology and Center for Neuroscience, University of Colorado at Boulder. Boulder, USA

## Abstract

The transitive inference (TI) task assesses the ability to generalize learned knowledge to new contexts, and is thought to depend on the hippocampus (Dusek & Eichenbaum, 1997). Animals or humans learn in separate trials to choose stimulus *A *over *B*, *B *over *C*, *C *over *D *and *D *over *E*, via reinforcement feedback. Transitive responding based on the hierarchical structure *A *> *B *> *C *> *D *> *E *is then tested with the novel *BD *pair. We and others have argued that successful *BD *performance by animals – and even humans in some implicit studies – can be explained by simple reinforcement learning processes which do not depend critically on the hippocampus, but rather on the striatal dopamine system. We recently showed that the benzodiazepene midazolam, which is thought to disrupt hippocampal function, profoundly impaired human memory recall performance but actually enhanced implicit TI performance (Frank, O'Reilly & Curran, 2006). We posited that midazolam biased participants to recruit striatum during learning due to dysfunctional hippocampal processing, and that this change actually supported generalization of reinforcement values. Greene (2007) questions the validity of our pharmacological assumptions and argues that our conclusions are unfounded. Here we stand by our original hypothesis, which remains the most parsimonious account of the data, and is grounded by multiple lines of evidence.

## Background

Our interpretation of our findings was based on the following premises. When humans are prevented from becoming explicitly aware of the hierarchy they can still perform better than chance at the novel "inference" test using an implicit reinforcement learning system [1]. Several mathematical and neural models show that when trained with the TI task procedure, stimulus *B *develops a higher associative strength then stimulus *D*, and that transitive responding can be achieved simply by comparing these implicit values [1-5]. Thus although under some circumstances the hippocampus can play a subtle modulatory role in setting up these values [4] (and is likely critical for genuine, explicit logical inference), neural models suggest that the elemental associative learning process itself does not depend on the hippocampus, but rather on the striatal dopamine system [6,7]. Moreover, the striatal and hippocampal systems often compete, such that disruption of one system can lead to enhanced performance of tasks that depend on the other (for review: [8,9]).

We therefore posited that midazolam would disrupt the hippocampus [10-18], thereby removing its normal inhibitory interaction with the striatum, and allowing that system to dominate. The resulting impaired hippocampal learning but enhanced implicit reinforcement encoding led to (i) impaired explicit memory recall; (ii) a pattern of learning in the initial training phase of the TI task that is a characteristic signature of associative models: greater performance on the end anchor pairs *AB *and *DE*, which can be solved purely on the basis of reinforcement values, and worse performance on the conjunctive inner pairs *BC *and *CD*; (iii) substantially enhanced generalization of reinforcement values in the transitive test [19]. These results support predictions from more abstract mathematical models in which conjunctive learning is detrimental to subsequent transitive responding [5]. Finally, in another probabilistic learning task associated with the striatal dopamine system [7], midazolam led to spared performance and only caused deficits in the very first few trials of the task [19], when the hippocampus is usually most active in such tasks [20], consistent with other accounts on the role of the hippocampus in the early acquisition of probabilistic learning [21].

In his critique, Greene argued that our theory entails three critical assumptions that are "required but not met" [22] (pp. 1–2):

1. that other areas critical to TI are not affected by midazolam administration, so only hippocampal deactivation can explain the effect;

2. that midazolam deactivates the hippocampus so that it no longer functions in a mnemonic capacity; and

3. that midazolam influences explicit but not implicit memory.

It is reasonable to question the assumptions of our logic. Nevertheless, on the whole we believe this criticism is misguided. First, there is ample evidence to suggest that hippocampal function is dramatically impaired by midazolam administration, and critically, no indication that the striatal reinforcement learning system – strongly predicted to support reinforcement-based generalization in this task – is negatively affected. Second, while it is certainly possible that areas other than the hippocampus are affected by the systemic drug, it is entirely unclear why this would result in an *enhancement *in TI performance as was observed [19]. In contrast, our theoretical framework clearly accounts for the effect, and is supported by other diagnostic converging data in our study. Third, the notion that midazolam impairs explicit memory substantially more than implicit memory, while well supported by psychological data, is actually not a critical assumption of our logic, which depends more specifically on striatal-dependent implicit associative learning rather than on implicit learning in general.

## Discussion

Below we elaborate these and other issues in response to Greene's three criticisms, and identify some new avenues for research to more directly address the question.

### Assumption 1: other areas critical to TI account for the effect

Greene takes issue with our suggestion that midazolam improved TI performance by deactivating the hippocampus [19], stating that "it is entirely possible that the effects of midazolam on the TI task are attributable to deactivations of areas other than the hippocampus". He cites PET evidence that midazolam inactivates some of the very same frontal and parietal regions that have been shown to be activated during explicit TI performance in other studies [23,24]. We are puzzled by this point, as it is difficult to imagine how deactivation of a region that is normally activated during TI would lead to enhanced performance. Admitting possible additional frontal cortical effects of midazolam, our key point was that thus far no studies implicate striatal deactivation following midazolam. Other evidence from Parkinson's patients and dopamine medication manipulations support predictions from computational models of the basal ganglia, which suggest that this system is involved in learning reinforcement associations in the TI task [7]. These data provide converging evidence for a role of striatal DA system in learning implicit associative values in the TI task.

We note here that Greene is undeniably correct that by administering a systemic drug one cannot know for certain that either the observed behavioral decrements in recall (amnesia) and the associated improvement in TI, are related specifically to hippocampal deactivation. However, our central hypothesis is that the critical brain system supporting reinforcement-based learning in the TI and other feedback learning tasks is the striatal dopamine system, and to the best of our knowledge there is no evidence to suggest this system is inhibited by midazolam, either at the neural level or in learning tasks that depend on this system. Moreover, there is some evidence in rats that the two main peptide markers of activity in the direct and indirect pathways of the basal ganglia (dynorphin and enkephalin; [25]) are actually substantially *enhanced *under systemic midazolam administration [26,27], as are striatal dopamine levels [28]. These effects could conceivably result from the removal of the normal inhibitory interaction with hippocampus, which have been observed under a variety of conditions in both animals and humans [8,9]. As one recent (non-pharmacological) example in humans, when dual task conditions were introduced to interfere with explicit memory, procedural learning normally correlated with hippocampal activation and associated declarative knowledge proceeded instead in a habitual fashion that correlated with striatal activation [29].

### Assumption 2: midazolam obliterates hippocampal function

Greene also questions whether midazolam disrupts hippocampal function at all. That is, despite the dense expression of benzodiazepene receptors in the hippocampus [30], some human neuroimaging studies fail to detect hippocampal deactivation under midazolam. While one study did show a dose-dependent hippocampal effect [15], others using low doses did not. However, the effects of drugs on BOLD activity are not straightforward. First, BOLD is a very indirect and imprecise measure of neural activity, whereas direct administration of midazolam in the hippocampus show robust disruptions of both neural activity and plasticity [10-13]. These findings are not restricted to local injections into the hippocampus. For example, systemic midazolam administration has been shown to decrease hippocampal cholinergic activity [14]. While this action should clearly interfere with hippocampal function and memory encoding, it is much less clear what effect, if any, this would have on BOLD activity – it could simply add noise to the hippocampal system. Indeed, this hippocampal noise hypothesis has been modeled to account for various detrimental memory encoding effects of midazolam [17]). It is similarly unclear how midazolam's inhibitory effects on hippocampal long term potentiation [18] – the central mechanism thought to give rise to hippocampal memories – would translate into BOLD.

Furthermore, Greene also asserts that our theory requires midazolam to "deactivate the hippocampus to the point that it no longer functions in a mnemonic capacity". This is clearly not the case – while the low dose used in our study robustly impaired memory encoding/recall, this memory was far from obliterated altogether. These findings are similar to other studies purporting that midazolam impairs hippocampal function and substantially degrades, but does not eliminate, episodic memory [16,17,31]. In contrast, hippocampal amnesics were substantially impaired at learning the training pairs, particularly the inner pairs that require conjunctive encoding, never reaching training criteria [32]. Thus although these amnesics were impaired at the TI probe test, these data are not meaningful given that they never acquired the relevant premises in the first place. In contrast, participants on midazolam were impaired initially at the conjunctive inner pairs relative to control subjects, but this deficit was subtle enough that they were able to eventually overcome it. Other studies show that both humans and animals with hippocampal damage are completely unimpaired at re-combining learned elemental reinforcement values to support generalization of learned behavior to novel situations [33,34].

### Assumption 3: midazolam impairs explicit but not implicit memory

Finally, Greene claims that our argument requires midazolam to affect explicit but not implicit memory, but that there are a few studies showing some impairments in implicit memory. We note that first, there is substantial evidence to suggest that midazolam has a far greater proportional impairing effect on explicit than implicit memory [35-38]. Other recent studies showed that the same experimental procedures (including midazolam dose) used in our study selectively abolished event-related brain potentials associated with recall, which had been previously linked to hippocampal function, without affecting other memory components [31]. However, the need for midazolam to spare implicit memories of all types is actually not a requirement of our theory at all. Indeed, we posit that midazolam impairs the conjunctive encoding of stimulus features in the hippocampus, and while this function is critical for several aspects of explicit memory, it can also support some aspects of implicit memory [39]. Thus again our argument relies on the notion that midazolam has not been shown to impair striatal-dependent memories, i.e. in so-called procedural learning tasks.

### Future studies

To help further resolve this controversy, midazolam effects on hippocampal activity ought to be examined in situations for which the hippocampus is thought to be critical according to the same computational principles used to motivate our original hypothesis. It would be particularly informative to explore the effects of midazolam on a contrast between conjunctive and elemental memory encoding in the TI or other task showing hippocampal sensitivity to conjunctions [40]. Indeed, Greene's own study [41] showed hippocampal activation for the conjunctive "inner pairs" in the TI task, and midazolam impaired acquisition of these same pairs in our study [19], and impaired conjunctive encoding in other tasks [39].

## Conclusion

Our original study was motivated by an explicit theoretical and neurocomputational framework for why this pattern should be observed, and is consistent with other psychological and mathematical analysis. In contrast, while legitimately questioning some of our assumptions, Greene provides no alternative explanation for the finding that midazolam substantially improved TI performance in our study. Until such an interpretation arises, with supporting data, the most parsimonious explanation of our data is that motivated by *a priori *theoretical predictions. Greene's commentary does reaffirm that alternative opinions are valid and that as is always the case, further research is necessary.

## Abbreviations

TI: transitive inference

## Competing interests

The author(s) declare that they have no competing interests.

## Authors' contributions

M.J.F., R.C.O. and T.C wrote the paper.
